# Surgical Treatment of Pediatric Dog-bite Wounds: A 5-year Retrospective Review

**DOI:** 10.5811/westjem.2021.9.52235

**Published:** 2021-10-27

**Authors:** Christine J. Lee, Ekaterina Tiourin, Sawyer Schuljak, Jonathan Phan, Theodore W. Heyming, John Schomberg, Elizabeth Wallace, Yigit S. Guner, Raj M. Vyas

**Affiliations:** * University of California – Irvine School of Medicine, Department of Plastic Surgery, Orange, California; † Children’s Hospital Orange County, Division of Plastic Surgery, Orange, California; ‡ University of California - Riverside, School of Medicine, Riverside, California; § University of California – Irvine Medical Center, Department of Surgery, Irvine, California; ¶ Children’s Hospital Orange County, Department of Emergency Medicine, Orange, California; || Children’s Hospital Orange County, Division of Pediatric Surgery, Orange, California; # University of California – Irvine School of Medicine, Department of Emergency Medicine, Orange, California; ** Children’s Hospital Orange County, Department of Nursing, Orange, California; †† Children’s Hospital Orange County, CHOC Research Institute, Orange, California

## Abstract

**Introduction:**

Dog bites are a significant health concern in the pediatric population. Few studies published to date have stratified the injuries caused by dog bites based on surgical severity to elucidate the contributing risk factors.

**Methods:**

We used an electronic hospital database to identify all patients ≤17 years of age treated for dog bites from 2013–2018. Data related to patient demographics, injury type, intervention, dog breed, and payer source were collected. We extracted socioeconomic data from the American Community Survey. Data related to dog breed was obtained from public records on dog licenses. We calculated descriptive statistics as well as relative risk of dog bite by breed.

**Results:**

Of 1,252 injuries identified in 967 pediatric patients, 17.1% required consultation with a surgical specialist for repair. Bites affecting the head/neck region were most common (61.7%) and most likely to require operating room intervention (P = 0.002). The relative risk of a patient being bitten in a low-income area was 2.24, compared with 0.46 in a high-income area. Among cases where the breed of dog responsible for the bite was known, the dog breed most commonly associated with severe bites was the pit bull (relative risk vs German shepherd 8.53, relative risk vs unknown, 3.28).

**Conclusion:**

The majority of injuries did not require repair and were sufficiently handled by an emergency physician. Repair by a surgical specialist was required <20% of the time, usually for bites affecting the head/neck region. Disparities in the frequency and characteristics of dog bites across socioeconomic levels and dog breeds suggest that public education efforts may decrease the incidence of pediatric dog bites.

## INTRODUCTION

With over 4.5 million dog bite injuries reported each year in the United States, dog bites continue to be a significant public health concern.^1^ Children are at high risk for dog bite injury, with many incidents reported at or near a victim’s home.^2^ The current global pandemic has necessitated virtual learning, and children are spending more time at home. The latest report from the US Centers for Disease Control and Prevention on the frequency of dog bites reported that 800,000 individuals sought medical attention for a dog-bite injury in 2001.^3^ These numbers are expected to surge due to stay-at-home guidelines during the current pandemic.

Many studies have identified trends in pediatric dog-bite injuries and interventions,^4^–^15^ but few studies have stratified injury severity based on the type of surgical treatment required. Significant damage to the face, which is the area most commonly affected in children who sustain dog bites, may require the specialized skills of a subspecialist who can reconstruct the complex functional and aesthetic components of the affected anatomy.^16^ In younger patients, delicate anatomy and limited compliance may require treatment in the operating room (OR), instead of a bedside procedure. The surgical approach is also determined by injury severity, which has previously been shown to be associated with socioeconomic factors in adults with dog bites.^17^ We sought to examine the interplay among these factors in pediatric patients who presented for treatment of dog-bite injuries at our institution.

Orange County, CA, where our institution resides, is the sixth largest county by population in the US, with many low-income and affluent communities in close proximity to one another. Our academic pediatric trauma center is the only pediatric hospital serving this diverse population of over three million. This makes our institution an ideal setting for an investigation of the etiology and treatment of pediatric dog-bite injuries. In this study, we describe our five-year experience and aim to characterize the settings in which a surgeon is required for the treatment of pediatric dog-bite injury. We also collected information from public records and healthcare databases to evaluate external risk factors that may increase risk for dog bites, such as socioeconomic status and breed of dog. Delineating the injury patterns in this high-risk population may both streamline care and guide future prevention efforts.

## METHODS

This was a retrospective cross-sectional study of all children aged 0 to 17 years treated for dog-bite injury during the period from 2013–2018 at our institution. The inclusion criteria were all pediatric patients presenting to the pediatric emergency department (ED) during the study period and identified in the electronic health record (EHR) as having an acute dog bite injury (*International Classification of Diseases*, *Ninth Revision* and *Tenth Revision, Clinical Modification* [*ICD-9*] E906.0 and *ICD-10-CM* W54.0). Exclusion criteria were bite wounds that had already received a procedure at another institution and transferred to our institution for delayed reconstruction, patients who presented > 24 hours after the injury, and any subsequent visits related to the same initial injury. Two unblinded abstractors were uniformly trained to use a pilot-tested, standardized, online data abstraction form with coding rules. Data abstraction was routinely monitored to ensure systematic data collection including refresher training and review of coding rules. We did not exclude records with missing data; missing values for categorical variables were documented as unknown.

**Population Health Research Capsule**

*Dog bites are a significant health concern in children. Most dog bites occur from age 1–5, and affect the head and neck region.*

What was the research question?

*Do sociodemographic factors and dog breed impact pediatric dog-bite injuries and their severity?*

What was the major finding of the study?

*Lower socioeconomic status increased risk for dog bites. Larger dogs were associated with more severe injury.*

How does this improve population health?

*This study informs injury prevention efforts that may target communities at risk including those with lower socioeconomic status.*

The descriptive features captured in this study included the following: sociodemographic information (age, race, gender, ethnicity, payer source, and median income associated with residence ZIP code); clinical variables (wound depth, wound diameter, level of intervention required, number of body sites wounded, and anatomical site of injury); and information on the dog (relationship to dog, breed of dog). Wound depth was categorized as superficial (partial thickness skin wounds, scratches, excoriations, dermabrasions), deep (full-thickness skin wounds without trauma to underlying tissue), and complex (full thickness wounds with trauma to underlying tissues such as tendons, nerves, vessels). Information on the dog breed, patient’s relationship to the dog, and location where the injury occurred were first abstracted from the provider notes in the EHR and then cross-referenced with information included in the Animal Bite Human Reporting Form sent to the county health department.

Socioeconomic data such as median income was extracted from the American Community Survey (ACS).^18^ We obtained county records of city-level dog populations from the county animal shelter.^19^ The relative proportions of various dog breeds in the county were applied to city-level estimates of dog population to determine the relative risk of dog bite. We further stratified the data analyzed for each dog breed based on bite severity and median income in the area where the dog bite occurred. A phylogenetic tree of dog breeds was constructed using data from the National Human Genome Research Institute Dog Genome project.^21^ We constructed the phylogenetic tree using a circular tree plot to visualize bite frequency across genetic groups.

### Statistical Analysis

We calculated the relative risk of being bitten by a specific breed of dog, the relative risk of being bitten in a lower-income area, and the relative risk of sustaining a severe, rather than moderate or mild, dog-bite injury. The relative risk of being bitten by a specific breed of dog was calculated using dog population data collected by the animal shelters of our county, which collect data for all licensed dogs in the county. We ranked dog breeds according to relative risk of bite, compared to the risk of being bitten by any member of the dog population in the county. The relative risk of dog bite was mapped onto each breed in the phylogenetic tree. If no bite data was observed for a specific dog breed, the relative risk was set to one.

We calculated *P*-values using the chi-square test for cell size >100 and Fisher’s exact test for cell size <100. In this study, the Fisher’s and chi-square *P*-values measured distribution of a given variable after stratification by another categorical variable, in comparison to the distribution of all other categories summed. For continuous measures such as bite diameter, a Wilcoxon rank-sum test was used to measure the difference in distribution among continuous measures. We used the R programming language to conduct these analyses. Income and dog-bite frequency were mapped using the *Choroplethr* package (R Foundation for Statistical Computing, Vienna, Austria).^20^

## RESULTS

**Figure 1:**
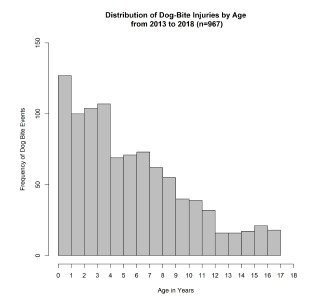
Distribution of dog bite injuries by age.

From 2013 to 2018, 967 pediatric patients at our children’s hospital were identified as victims of a dog bite. The mean and median ages of pediatric patients who sustained dog-bite injuries were six years and five years, respectively. The mode of the age variable in this study was three years. After stratification into age categories of 1–5 years, 6–10 years, and >10 years of age, the 1–5 age group was identified as the group of patients that made up the greatest proportion of those bitten (53.4%). The risk for dog-bite injury was inversely correlated with age, with a Pearson correlation coefficient of −0.76 (Figure 1). Regardless of age, children are bitten most frequently by a dog living in their own home (33.4%), followed by pets belonging to family and friends (22.4%) (Supplemental Table).

Our analysis of the sociodemographic data collected revealed that the racial distribution of pediatric patients who sustained dog-bite injuries was similar to the racial make-up of the community, with 64.6% of patients in the study identifying as White/Caucasian. It should be noted that patient families identifying as Latino were disproportionately represented in this survey. The 2017 ACS reported that 34.2% of the residents in the county identified as Latino, while 55.2% of the patient population in this study identified as Latino (with only 1.16% of study participants refusing to answer this question). It should also be noted that a large proportion of the patient families included in this study were covered by Medicare (22.4%) or Medicaid (29.5%); 41.4% were covered by private insurance, and the remaining 6.6% were self-pay (Table 1).

### Level of Intervention

Most injuries did not require specialist or OR services; 71.8% of bites did not require wound repair, while 17.1% of patients required specialist consultation for wound repair in the ED or the OR. The distribution of bite severity mirrored this pattern, with 70.5% of bites classified as “superficial” (partial thickness, scratches, excoriations, abrasions); 21.1% of bites classified as “deep” (full thickness without trauma to underlying structures); and 8.5% of bites classified as “complex” (full thickness with trauma to underlying structures such as tendons, nerves, and/or vessels). Analysis of the data to determine which anatomical area was most commonly affected revealed that 61.7% of bites were inflicted on the head or neck, 20.6% on the hands or arms, and 13.0% on the feet or legs (Table 2).

**Figure 2:**
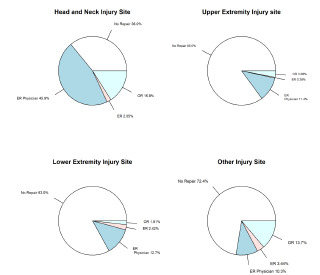
Level of intervention by anatomic site of injury.

When we investigated the relationship between anatomical site of injury (head, upper extremity, lower extremity, other) and type of intervention (no repair, emergency physician repair [EP], surgical specialist repair in ED, specialist repair in OR), we found that head and neck injuries were significantly more likely to require repair (*P* = <.0001). When stratifying injuries by different levels of repair (EP, surgical specialist in ED, and specialist repair in OR) there were statistically significant differences in the proportion of observed injuries across different anatomic sites. The largest difference in proportion was observed in head and neck injuries, which contributed to 41.2% of cases not requiring repair, and 86.2%, 69.6%, and 88.5% to cases requiring repair by EPs, surgical specialists in the ED, and repair performed by specialists in the OR, respectively. This association persisted even when “no repair” patients were removed from the dataset (*P* = 0.002). This data is presented in Table 3 and Figure 2.

When we examined the association between requirement for surgical treatment and bite severity, the data showed that 82.3% of complex wounds (full thickness with trauma to underlying structures such as tendons, nerves, and/or vessels) were treated in the OR, 9.8% of complex wounds were treated by a specialist in the ED, and 1.9% of wounds were repaired by a general EP. This observed pattern contrasted with that observed for deep wounds (full thickness without trauma to underlying structures), for which the majority (79.4%) were treated by an EP. The majority of superficial wounds (76.3%) required no repair.

### Socioeconomic Status

**Figure 3:**
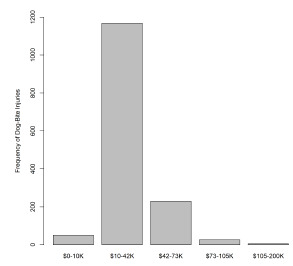
Distribution of dog bite injuries by level of annual income.

**Figure 4:**
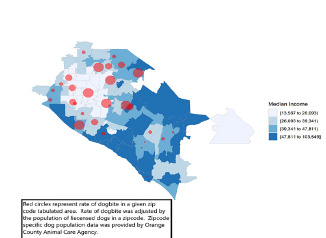
Distribution of dog bite injuries based on zip code and median income quartile.

We used ZIP codes to map city-level reports of median income from the ACS. The ZIP code was used to approximate the economic status of a patient family to evaluate the association between economic status and the frequency of bites. According to the 2017 ACS, the median income in the county is $89,000. Analysis of the study data showed that 67.9% of patients lived in areas with median annual income greater than $42,000, and 32.1% of patients lived in areas with median income of $42,000 or less (Figure 3). Using population-based estimates of the total dog population for each area, the relative risk of a pediatric patient being bitten in a low-income area (median income ≤ $42,000) was 2.24-fold greater than the baseline risk of being bitten in the county. In contrast, the relative risk of a pediatric patient being bitten in a high-income area (median annual income > $42,000) was 0.46. The relative proportion of biting dogs in the general dog population was significantly greater in low- vs high-income areas (*P* <.0001). These differences are illustrated in Figure 4; there was a significant difference in the proportion of dogs inflicting bites in neighborhoods with median income <$42,000 compared to the proportion of dogs inflicting bites in neighborhoods with median income >$42,000.

**Figure 5:**
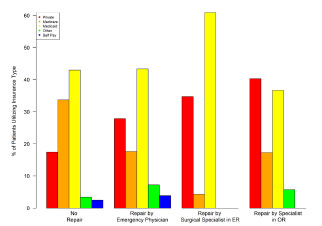
Level of intervention by insurance payer group.

We performed an analysis of the distribution of bites across insurance payer and level of intervention, with insurance status used as a proxy for economic status. Patients who used private insurance to pay for hospital services were significantly more likely to receive treatment by a specialist or treatment in the OR than patients who used Medicaid or Medicare to pay for hospital services (*P* <.0001) (Table 3). Medicaid patients accounted for only 15% of those with injuries treated by specialists. Among those who received OR treatment for dog bites, 75% used Medicare or private insurance to pay for hospital services (Figure 5).

### Dog Breed

**Figure 6:**
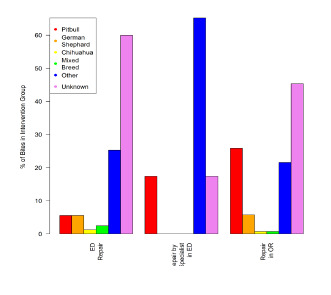
Level of intervention by breed of dog.

In 61.4% of cases included in the study, the breed of the dog that had bitten a particular patient was unknown. Among the cases where the breed of the dog responsible for the injury was reported, representation was as follows: Chihuahua mix, 7%; pit bull mix, 7.6%; German shepherd mix, 3.3%; other or mixed breed, 20.4%. No significant relationship was found between dog breed and anatomical site of injury, or between dog breed and median income in the area where the dog bite occurred. There was, however, a significant association between breed and the requirement for surgical treatment by a specialist (Table 4). The likelihood that the patient had been bitten by a pit bull increased as the level of intervention increased from no repair (6.0%) to repair in the OR (25.8%) (Figure 6).

**Figure 7:**
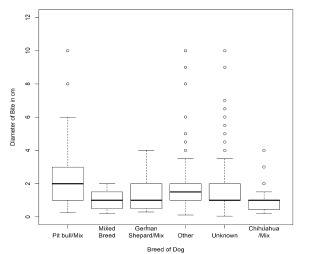
Distribution of bite diameter by breed of dog.

Dog breed was a significant predictor of bite severity (*P* <.0001) and of bite diameter (*P* <.0001). Pit bull bites were found to be significantly larger, deeper, and/or more complex than the average dog bites included in this study (Figure 7). Patients included in this study were more than four times as likely to have been bitten by a pit bull than by a German shepherd, and more than twice as likely to have been bitten by a pit bull, when compared with a dog of unknown breed. Furthermore, the relative risk of a pit bull inflicting a complex (full thickness with trauma to underlying structures) or deep (full thickness without trauma to underlying structures) bite was 17 times that observed for non-pit bull dogs. The relative risk of a German shepherd inflicting a complex or deep bite was 2.66, and the relative risk that a dog of unknown breed would inflict a complex or deep bite was 0.23. The relative risk of being bitten by a pit bull did not differ greatly between high-income cities and low-income cities, with relative risk of 8.06 and 8.17, respectively (Table 5).

**Figure 8:**
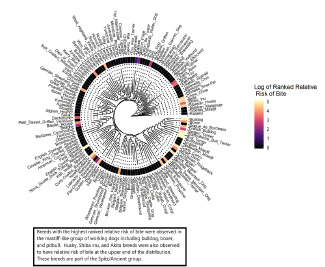
Phylogenetic tree of dog breeds associated with risk for dog bite within the county.

We constructed a phylogenetic tree of dog breeds to identify clades with an increased relative risk of bite, compared to the general dog population (Figure 8). This visualization revealed increased relative risk for dog bite in dog breeds designated as “working dogs” by the American Kennel Club. The breeds in this group associated with high relative risk for bite-related injury were bulldog, boxer, French bulldog, pit bull, mastiff, Great Dane, Rottweiler, and Doberman pinscher. Siberian husky, chow chow, and Akita breeds also had increased risk of dog bite compared to the general population of dogs in the county. This latter group of dogs is classified on the side of the canine phylogenetic tree most distant from dogs classified as “working dogs.” Among all dogs within the phylogenetic tree, husky, chow chow, and Akita breeds are most closely related to the common ancestor of all canines, the wolf. Although the husky is classified as a working dog, it is not closely related to the clade of working dogs listed above. The dogs with decreased relative risk of bite (basset hound, beagle, and dachshund) were clustered in a group of dogs classified by the American Kennel Club as hounds (relative risk, < 1.00).

## DISCUSSION

Dog bite injuries continue to be prevalent in the pediatric population, especially among young children. Similar to previous studies,^4^,^5^,^7^–^9^ our analysis showed that the majority of dog bites in our study affected children 1–5 years of age, with risk for dog bite decreasing as age increased. Dogs may perceive the behavior of young children as threatening.^22^–^24^ Infants, toddlers, and preschool children are less cautious, tend to explore their environments with their hands and mouths, and exhibit unpredictable behaviors, such as suddenly kissing, biting, grabbing, and climbing upon a dog. Because of its proximity to the floor, the head and neck region of children is particularly susceptible to dog-bite injury; in adults, the extremities are most susceptible.^17^ Our analysis supports prior studies^4^,^5^,^11^,^12^ demonstrating that the majority of dog bites in children affect the head and neck region (61.7%), followed by the hand or arms (20.6%).

A previous study by our group of dog-bite injuries in the county showed that 60% of dog bites in adult patients received no intervention.^17^ Because the facial region is frequently involved when a dog bites a child, the families of children with dog-bite injuries are also more likely to seek medical attention than adults who have sustained dog bites.^25^ Pediatric patients may, therefore, be more likely to present to the ED with superficial dog-bite injuries,^6^ which may partially account for the increased incidence of reported dog bites in children compared to adults. Of the pediatric patients who presented to the ED at our institution during the study period, 71.9% required no intervention because their injuries were superficial. Of the pediatric patients in our study requiring intervention, the greatest proportion of dog-bite injuries that necessitated repair in the OR affected the head and neck areas. Dog-bite injury to the facial region not only threatens function but may also have a lasting impact on physical appearance as the child grows into adulthood. The complex nature of head and neck physiology and anatomy, therefore, often merits consultation with a specialist and intervention in the OR.^15^,^26^ In our study, complex and deep injuries with larger diameters were likely to require specialist intervention.

Our analysis goes further to reveal how socioeconomic factors influence the management of dog-bite injury. A median annual income below $42,000 conferred a 2.24 relative risk for pediatric dog-bite injury, compared to a 0.46 relative risk in regions with high median annual income. This trend is consistent with the findings of a study by Ruiz-Casares et al, which demonstrated that children in low-income families are the most vulnerable to unintentional injury.^27^ Parents in low-income households may need to attend to work obligations and may, therefore, be unavailable to supervise young children and without the means to pay for daycare services. Young children supervised by older siblings have increased risk for injury, compared to young children supervised by their parents.^28^,^29^ Because adults are generally able to protect themselves, the risk for dog bite and associated patterns of injury in adults does not seem to be impacted by annual income.^17^ Furthermore, dogs in low-income households are less likely to be supervised, less likely to be sufficiently trained, and less likely to be kept in an area enclosed by fencing or gates.^30^ Low-income households are also more likely to have large-breed dogs for protective purposes.^30^ This combination of inadequate resources for child supervision and large-breed dogs without robust training may account for the increased incidence of pediatric dog-bite injury in low-income households.

In our analysis, insurance type was used as an index for socioeconomic status. Our study shows that children in families with Medicaid or self-pay status were more likely to experience a dog-bite injury, but less likely to have their injuries repaired by specialists in the OR. It is unclear whether the difference in service utilization between private insurance payers vs Medicaid or self-payers reflects systemic obstacles or, rather, a parental preference for ED intervention based on financial concerns. While Essig et al showed that the surgical management of pediatric facial dog-bite injuries by specialists in either the ED or OR had no significant effect on the risk for surgical-site infection or reoperation,^31^ it would be interesting to study the outcomes of dog-bite injuries treated by ED clinicians, compared with similar injuries that were treated by surgical specialists. The results of a comparative cohort study might reveal whether treatment by a specialist decreased the incidence of infection, scarring, or later return to the OR. A significant difference in the outcomes of pediatric dog-bite injury with specialist vs non-specialist treatment might ultimately result in a change in treatment patterns and improved public health.

Many studies have attempted to elucidate the role of dog breed in bite injuries. In the literature the dog breeds most commonly associated with pediatric dog-bite injuries include the pit bull, Rottweiler, German shepherd, terrier, and mixed.^9^,^10^,^32^ In our analysis, German shepherds were responsible for the highest number of pediatric dog-bite injuries, but pit bulls were responsible for the most severe injuries. In a related study conducted at a Level I pediatric trauma center, Alizadeh et al showed that 47.8% of pediatric dog bites that involved a pit bull required surgical intervention.^33^ Many studies have reported similar results of pit bull-related aggression, and this particular breed has been considered a public health risk; several countries and US cities have introduced breed-specific bans.^34^,^35^

It should be noted that aggressive canine behavior is multifactorial, with genetic as well as human interference-related contributing factors.^36^,^37^ However, breed-specific legislation has been criticized for being ineffective, difficult to implement, and harmful to the welfare of dogs. Breed-specific bans may also be based on incomplete data from health records or sensationalized media reports.^8^,^38^,^39^ We agree that rather than breed-specific laws, efforts to decrease the frequency of pediatric dog-bite injury should focus on identifying the precipitating factors. Clinicians should be educated to include as part of their history questions about whether the child presenting for care was supervised and whether the dog was partitioned from the child, in addition to questions about the age, gender, breed, and level of training of the dog. A more complete health record would increase the accuracy of the data related to dog-bite injury in pediatric patients.

## LIMITATIONS

There were several limitations to our study. The socioeconomic data that we extracted from the ACS was not a true measure of family income, as these pooled data represent neighborhood-level rather than individualized patient information. The data presented in this analysis is specific to a high-volume, academic healthcare institution that serves a large and diverse community. The findings may, therefore, not be generalizable to all institutions and populations. We did not stratify the data used for analysis based on surgical subspecialty or type of dog-bite injury. Not all bites could be attributed to a specific breed or mixed breed of dog. As a result, the relative risk of bite in some breeds may have been under-reported. Additional bias may occur in breeds with small reported populations in the community; these breeds may have instability in the estimates of relative risk of bite due to small samples that are not representative of a given dog breed.

Additional studies will be designed to elucidate whether plastic surgeons, otolaryngologists, or general surgeons are more frequently involved with certain types of pediatric dog-bite injuries. Such an investigation would help to streamline workflow and to increase the use of a multidisciplinary approach in pediatric EDs. With interest, we continue to monitor and study how trends in the etiology and management of pediatric dog-bite injuries may change as social distancing alters the way that children interact with their environments.

## CONCLUSION

Our findings support previous reports that pediatric dog-bite injuries occur more frequently in children aged 1–5 years. Most dog-bite injuries in this study were caused by encounters with large dogs, and bites from pit bulls were associated with significantly more severe injury. The anatomical site affected most commonly was the head and neck region. The dog-bite injuries that most frequently require subspecialist surgical intervention are those affecting the head and neck region and those involving extensive soft tissue damage. Low socioeconomic status may increase the risk of dog-bite injury. Pediatric patients with private health insurance were more likely than others to receive surgical intervention for dog-bite injuries.

## Supplementary Information



## Figures and Tables

**Table 1:** Characteristics of pediatric dog-bite victims who presented to the emergency department from 2013–2018.

Characteristics	Frequency n (%)*
Number of patients	943
Age (year)	
Mean	6.04
Median	5
Mode	3
Gender	
Female	408 (43.2%)
Male	535 (56.7%)
Race	
White	610 (64.6%)
Black	12 (1.3%)
Asian	55 (5.8%)
American Indian	2 (0.2%)
Hispanic	19 (2.0%)
Native Hawaiian	8 (0.8%)
Other	215 (22.7%)
Refused	19 (2.0%)
Ethnicity	
Not Latino	421 (44.6%)
Latino	521 (55.2%)
Refused	11 (1.2%)
Payer	
Medicare	212 (22.4%)
Medicaid	279 (29.5%)
Private	391 (41.4%)
Self-pay	40 (4.2%)
Other	21 (2.2%)

* Frequencies reported are limited to all patients with clinical and demographic data.

**Table 2:** Characteristics of dog-bite injuries.

Characteristics	Frequency n (%)
Number of injuries	1,252
Level of intervention	
No repair	677 (54.0%)
Repair by EP	413 (32.9%)
Repair by specialist in ED	23 (1.8%)
Repair in OR	139 (11.1%)
Depth^*^	
Superficial	861 (70.5%)
Deep	258 (21.1%)
Complex	102 (8.3%)
Anatomic site	
Head/neck	774 (61.7%)
Upper extremity hand	153 (12.2%)
Upper extremity arm	105 (8.4%)
Lower extremity foot	18 (1.4%)
Lower extremity leg	145 (11.6%)
Other	57 (4.5%)

The depth variable was incomplete; thus, the percentages represent the number of injuries within each depth category out of the total number of injuries with complete wound depth data (n = 1,221)

**Table 3:** Level of intervention by injury location and payer source.

Characteristic	No repair	Repair by EP	Repair by surgical specialist in ED	Repair by specialist in OR
Total	677	413	23	139
Injury Location				
Head/neck	279 (41.2%)	356 (86.2%)	16 (69.6%)	123 (88.5%)
Upper extremity arm	141 (20.8%)	12 (2.9%)	0(0%)	0 (0%)
Upper extremity hand	80 (11.8%)	18 (4.4%)	1 (4.4%)	6 (4.3%)
Lower extremity leg	17 (2.5%)	0 (0%)	1 (4.3%)	0 (0%)
Lower extremity foot	118 (17.4%)	21 (5.1%)	3 (13.0%)	3 (2.2%)
Other	42 (6.2%)	6 (1.5%)	2 (8.7%)	7 (5.0%)
P-value	<.0001			
Payer source				
Medicare	114 (16.8%)	109(26.4%)	8 (34.8%)	54 (38.8%)
Medicaid	227 (33.5%)	71 (17.2%)	1 (4.3%)	24 (17.3%)
Private	288 (42.5%)	174 (42.1%)	11 (47.8%)	51 (36.7%)
Self-Pay	23 (3.4%)	26 (6.3%)	0 (0%)	6 (4.3%)
Other	17 (2.5%)	12 (2.9%)	0 (0%)	0 (0%)
P-value	<.0001			
Wound severity				
Superficial	657 (98.5%)	188 (47.5%)	9 (39.1%)	7 (51.4%)
Deep	4 (0.5%)	205 (51.8%)	4 (17.3%)	45 (33.0%)
Complex	6 (0.8%)	2 (0.5%)	10 (43.4%)	84 (61.7%)
P-value	0.0001			

*EP*, emergency physician; *ED*, emergency department; *OR*, operating room.

**Table 4:** Dog-bite visits by breed of dog from 2013–2018.

Characteristic	Pit Bull	Mixed Breed	German Shepherd	Other	Unknown	Chihuahua
Body region injured						
Head/neck	60(57.6%)	20(74.0%)	34(70.8%)	172(66.6%)	449(60.8%)	37(49.3%)
Upper extremity (hand/arm)	22(21.1%)	2(7.4%)	9(18.7%)	54(20.9%)	150(20.3%)	20(26.6%)
Lower ectremity (leg/foot)	14(13.4%)	2(7.4%)	2(4.2%)	23(8.9%)	110(14.9%)	12(16.0%)
Other	8(7.7%)	3(11.1%)	3(6.3%)	9(3.5%)	29(3.9%)	6(8%)
P-value	0.4	0.34	0.19	0.09	0.07	0.08
Median Income by city reported by ACS						
<$42,000/year	23(32.3%)	7(38.8%)	10(32.2%)	53(30.8%)	191(33.2%)	16(23.5%)
>$42,000/year	48(67.6%)	11(61.1%)	21(67.7%)	119(69.1%)	384(66.7%)	52(76.4%)
P-value	1	0.6	1	0.71	0.38	0.13
Level of intervention						
No repair	41(39.4%)	16(59.2%)	17(35.4%)	109(42.2%)	424(57.4%)	69(92.0%)
Repair by EP	23(22.1%)	10(37.0%)	23(47.9%)	104(40.3%)	247(33.4%)	5(6.6%)
Repair by surgical specialist in ED	4(3.8%)	0(0%)	0(0%)	15(5.8%)	4(0.5%)	0
Repair by specialist in OR	26(34.6%)	1(3.7%)	8(3.7%)	30(11.6%)	63(8.5%)	1(1.3%)
P-value	0.001	0.69	0.01	<.0001	0.004	<.0001

*ACS*, American Community Survey; *EP*, emergency physician; *ED*, emergency department; *OR*, operating room.

**Table 5:** Relative risk* of bite by dog breed using estimated dog population.

Dog breed	Number of bite events attributed to breed	Proportion of bites in database attributed to breed	Estimated population of dog breed in county	Proportion of bite events with >1 body site bitten	Average bite diameter in cm	RR of breed biting compared to general dog population	RR of inflicting deep or complex wound	RR of bite occurring in a low median income city	RR of bite occurring in a high median income city
Pit bull	75	7.75%	34,464(2.90%)	32.67%	2.9	8.53	17.07	8.17	8.06
German shepherd	32	3.30%	142,60(1.20%)	16.00%	1.62	2.02	2.66	1.97	1.95
Chihuahua mix	68	7.03%	534,78(4.50%)	29.10%	0.99	3.35	0.51	2.46	3.78
Mixed breed	18	1.86%	118,841(10.0%)	44.40%	0.95	0.2	1.7	0.24	0.18
Cocker spaniel	7	0.72%	142,60(1.20%)	28.57%	1.21	0.53	0.74	1.66	0*
Other breed	180	18.6%	818,817(68.9%)	36.20%	1.817	0.27	0.23	0.25	0.27
Unknown breed	593	61.3%	133,102(11.2%)	19.07%	1.63	3.28	2.5	3.3	5.39

* All relative risks in comparison to rate observed in general dog population.

*RR*, relative risk; *cm*, centimeter.
