# Supplementary material for: Surgical Treatment of Pediatric Dog-bite Wounds: A 5-year Retrospective Review
Source: West J Emerg Med. 2021 Oct 27;22(6):1301–10. doi: 10.5811/westjem.2021.9.52235 (PMC8597704; doi:10.5811/westjem.2021.9.52235)
Supplement: Supplementary file 1 [file wjem-22-1301-s001.docx]

**Supplemental Table.** Distribution of pediatric dog bites across relationship and age strata.

| **Relationship** | **n(%)** | **Age 0-5** | **Age 5-10** | **Age >10yo** |
| --- | --- | --- | --- | --- |
| Pet living at home | 323 (33.4%) | 184 (36.2%) | 90(29.9%) | 49(30.8%) |
| Pet belonging to family/friends | 217 (22.4%) | 129 (25.4%) | 58 (19.2%) | 30 (18.8%) |
| Neighborhood pet | 121 (12.5%) | 48 (9.46%) | 51 (16.9%) | 22 (13.8%) |
| Other | 132 (13.6%) | 60 (11.8%) | 43 (14.2%) | 29 (18.2%) |
| Unknown | 174 (17.9%) | 86 (16.9%) | 59 (19.6%) | 29 (18.2%) |
| Total | 967 (100%) | 507 (52.4%) | 301 (31.1%) | 159 (16.4%) |

*yo*, years old.
